# Genomic profile predicts the efficacy of neoadjuvant chemotherapy for cervical cancer patients

**DOI:** 10.1186/s12885-015-1703-1

**Published:** 2015-10-19

**Authors:** Naoki Horikawa, Tsukasa Baba, Noriomi Matsumura, Ryusuke Murakami, Kaoru Abiko, Junzo Hamanishi, Ken Yamaguchi, Masafumi Koshiyama, Yumiko Yoshioka, Ikuo Konishi

**Affiliations:** Department of Gynecology and Obstetrics, Kyoto University Graduate School of Medicine, 54 Shogoin Kawahara-cho, Kyoto, Sakyo-ku 606-8507 Japan

**Keywords:** Neoadjuvant chemotherapy, Cervical cancer, Bioinformatics, Glutathione pathway, *UGT1A1* polymorphism

## Abstract

**Background:**

Neoadjuvant chemotherapy (NAC) using platinum and irinotecan (CPT-11) followed by radical excision has been shown to be a valid treatment for locally advanced squamous cervical cancer (SCC) patients. However, in NAC-resistant or NAC-toxic cases, surgical treatment or radiotherapy might be delayed and the prognosis may be adversely affected. Therefore, it is important to establish a method to predict the efficacy of NAC.

**Methods:**

Gene expression microarrays of SCC tissue samples (*n* = 12) and *UGT1A1* genotyping of blood samples (*n* = 23) were investigated in terms of their association with NAC sensitivity. Gene expression and drug sensitivity of SCC cell lines were analyzed for validation.

**Results:**

Microarray analysis revealed that the glutathione metabolic pathway (GMP) was significantly up-regulated in NAC-resistant patients (*p* < 0.01), and there was a positive correlation between 50 % growth inhibitory concentrations of CPT-11 and predictive scores of GMP activation in SCC cells (r = 0.32, *p* < 0.05). The intracellular glutathione (GSH) concentration showed a highly positive correlation with GMP scores among 4 SCC cell lines (r = 0.72). *UGT1A1* genotyping revealed that patients with *UGT1A1* polymorphisms exhibited significantly higher response rates to NAC than those with the wild-type (79.5 vs. 49.5 %, respectively, *p* < 0.05).

**Conclusions:**

These results indicate that GMP scores of cancerous tissue combined with *UGT1A1* genotyping of blood samples may serve as highly potent markers for predicting the efficacy of NAC for individual SCC patients.

**Electronic supplementary material:**

The online version of this article (doi:10.1186/s12885-015-1703-1) contains supplementary material, which is available to authorized users.

## Background

Despite the prevalence of screening and advancement of therapy, the mortality rate among women of reproductive age due to cervical cancer has increased over the last two decades in Japan [1]. As locally advanced cervical cancer (LACC) of FIGO stage Ib2 or IIb is frequently accompanied by lymph node metastasis, patients bearing LACC, treated only with excision or radiation, exhibit a high incidence of recurrence, resulting in a poor survival outcome [2]. Thus, radical hysterectomy (RH) coupled with platinum-based chemotherapy or radiation and concurrent chemo-radiation therapy are now employed as intensive treatments for LACC [3, 4]. Neoadjuvant chemotherapy (NAC) prior to RH is usually conducted to reduce the tumor volume and improve the safety and integrity of surgery, but the prognosis of NAC-refractory patients worsens with the delay of the main treatment [4]. Therefore, to optimize the efficacy of NAC-RH, a method is needed to exclude chemo-refractory cases before starting initial therapy.

Recently, genomic characterization by analyzing gene expression microarrays or genotyping onco-related/suppressive genes has been developed to evaluate characteristic profiles of chemo-refractory tumors and host patients [5, 6]. Single-sample Gene Set Enrichment Analysis (ssGSEA) is a bioinformatics method to characterize the biological features of individual samples as signature scores based on gene expression microarrays [7]. It was reported that the *TP53* pathway ssGSEA score can be used to predict the response of lung cancer to radiation [8], but there has been no report suggesting that ssGSEA is useful to predict the chemo-susceptibility of LACC. On the other hand, it is well-known that irinotecan (CPT-11) causes severe side effects more frequently in patients with *UGT1A1* polymorphisms than the wild-type [9], and, thus, *UGT1A1* genotyping is a prerequisite before initiating chemotherapy using CPT-11 in a clinical setting. However, it remains unclear whether CPT-11 treatment is more effective in LACC patients with *UGT1A1* polymorphisms. In this study, we assessed whether the chemo-susceptibility of LACC could be evaluated based on tumor expression microarray analysis and host *UGT1A1* genotyping, in order to optimize the efficacy of NAC-RH.

## Methods

### Sampling and intervention

A total of 209 cervical cancer patients underwent primary therapy in the 5 years between 2007 and 2012. NAC-RH was administered to patients with stage Ib2 tumors larger than 4 cm or stage IIb tumors who did not desire radiotherapy, with 38 of the 209 patients meeting this criterion. The clinicopathological characteristics of these 38 LACC patients treated with NAC-RH from 2007 to 2012 at Kyoto University Hospital are summarized in Table 1. Peripheral blood samples from 23 patients were collected before the operation and their genomic DNA was extracted using a QIAamp blood kit (QIAGEN, Tokyo, Japan). Cancerous tissues were obtained from patients during the surgery, and their RNA was extracted using the RNeasy Mini Kit (QIAGEN). The RNA integrity number (RIN) was assessed with a bio-analyzer, and 12 samples with an RIN above 7.0 underwent further gene expression analysis (Additional file 4: Table S1). All materials were obtained following the receipt of written consent and used based on protocols approved by the Kyoto University Institutional Review Board. All patients received 1 to 3 courses of the combined therapy of CPT-11 and Nedaplatin (NDP) every 3 weeks before undergoing the surgery, as previously described: CPT-11, 60 mg/m^2^ on days 1 and 8; NDP, 60 mg/m^2^ on day 1 [10]. After the surgery, a total of 31 patients received 3 to 4 courses of CPT-11/NDP, 6 patients underwent other treatments because of CPT/NDP resistance, and 1 patient declined postsurgical treatment. Magnetic resonance imaging (MRI) was conducted before initiating chemotherapy and after the completion of each course until surgery. The tumor shrinkage rate was calculated based on the largest diameter of the target lesion on MRI according to the response evaluation criteria in solid tumors (RECIST) [11]. Patients underwent modified Okabayashi’s RH [12] at the point of achieving favorable or improbable responses after 1 (*n* = 4), 2 (*n* = 33), or 3 (*n* = 1) courses of chemotherapy. Adverse events during NAC were evaluated according to the Common Terminology Criteria for Adverse Events [13].

**Table 1:** Characteristics of LACC patients treated with NAC followed by RH

Characteristics	Number	%
Total patients	*38*	
Median age (range)	49 (25–69)	
Performance status 0	38	100
FIGO stage		
1B2	12	31.6
2B	26	68.4
Pathology		
Squamous	36	94.7
Adenosquamous	2	5.3
Primary tumor size		
>4 cm	30	78.9
≦4 cm	8	21.1
Tumor response after NAC		
CR	5	13.2
PR	24	63.1
SD	8	21.1
PD	1	2.6
Shirinkage rate		
>50 %	25	65.8
≦50 %	13	34.2
Lymph node metastasis		
negative	23	60.5
positive pelvic	15	39.5
positive aortic	4	10.5
Recurrence	10	26.3

*CR* Complete response, *PR* Partial response, *SD* Stable disease, *PD* Progressive disease

### Cell lines and culture

Human cervical cancer cell lines: Ca-ski, SKGIIIa, Hela, and ME-180, were obtained from Riken BioResource Center (Tsukuba, Japan) and maintained in RPMI1640 (Nacalai Tesque, Kyoto, Japan) and DMEM (Gibco, Grand Island, NY, USA) supplemented with 10 % heat-inactivated fetal bovine serum (v/v; Biowest, France) and penicillin–streptomycin (100 IU/mL penicillin, 100 μg/mL streptomycin; Nacalai Tesque). All of them are representative cervical cancer cell lines, and their gene expression microarray data could be obtained with IC50 values for CPT-11 from the COSMIC dataset.

### Microarray analysis

Transcriptional gene expression microarrays were generated from 12 cervical cancer samples using U133 Plus 2.0 gene chips (Affymetrix, Santa Clara, CA, USA), and Robust Multi-Array Average (RMA) normalization was performed using R (version 2.15.1). Microarray data can be obtained at the Gene Expression Omnibus website (GSE70035, http://www.ncbi.nlm.nih.gov/geo/). Probes showing expression values >5.0 in at least one sample and standard deviation >0.2 across all samples were filtered to perform gene expression analysis with differentially expressed genes, and the SAMROC method [14] was used for statistical analysis, as previously described [15]. Gene Set Enrichment Analysis (GSEA) was performed using the Molecular Signatures Database (http://www.broad. mit.edu/gsea/msigdb/index.jsp). A variant of GSEA, single-sample GSEA (ssGSEA), was performed using R to predict gene signature activity in squamous cell carcinoma (SCC) cells based on the Catalogue Of Somatic Mutation In Cancer (COSMIC, http://cancer.sanger.ac.uk/cosmic) and HCT116 cells, colon cancer cell lines, web-published at Array Express: E-MEXP-1171, as well as our own samples.

### UGT1A1 genotyping and glutathione assay

The Invader *UGT1A1* Molecular Assay Kit (Third Wave Technologies, Madison, WT, USA) was used to detect UGT1A1*6 and UGT1A1*28 polymorphisms of genomic DNA derived from blood samples. Regarding cell line samples, the polymerase chain reaction (PCR) was carried out to amplify the characteristic regions using designed primers (Greiner Bio-One, Tokyo, Japan), and *UGT1A1* polymorphisms were determined by direct sequencing, as previously described [16].

Primers: UGT1A1*28 forward: 5’-TATA GTCACGTGACACAGTC’-3 and reverse: 5’-CCACTGGGATCAACAGTATCT’-3, UGT1A1*6 forward: 5’-AAGTAGGAGAGGGCGAACC’-3 and reverse: 3’-GTGGGCAGAACAGGTACT’-3.

Total GSH concentrations in cancer cells were assayed using the total GSH Quantification Kit (Dojindo Laboratories, Kumamoto, Japan) according to the manufacturer’s protocol.

### Statistical analyses

Group comparisons were made using the Mann–Whitney U test or Fisher’s exact test. Prognostic analyses were performed using the Log-rank and Cox proportional hazard tests. All statistical analyses were conducted using R software. Two-side probability values below 0.05 were considered significant.

## Results

### Characteristics of patients treated with NAC-RH

Clinical characteristics of the 38 LACC patients treated with NAC-RH are listed in Table 1 (median age: 49 years old, stage Ib2: *n* = 12, IIb: *n* = 26). Among these patients, 29 (76.3 %) exhibited a complete or partial response to NAC, and the tumor shrinkage rate exceeded 50 % in 25 patients. However, post-NAC pathological findings revealed node metastasis in 19 patients, resulting in recurrence in 10 patients. In DFS analysis (Table 2), the age, tumor size, and serum SCC values before treatment were not significant prognostic factors. Known major risk factors of recurrence, stage and node metastasis, were not determinants in this study, but lymphovascular invasion (LVSI) and a tumor shrinkage rate below 50 % exhibited significant differences regarding DFS (*p* < 0.05, Table 2). The Cox proportional hazard test revealed that a tumor shrinkage rate below 50 % was an independent risk factor (RR: 12.14, *p* < 0.05, Table 2 and Additional file 1: Figure S1), and patients with a rate < 50 % were defined as non-responders for further analysis.

**Table 2:** Univariate and multivariate analyses of factors predicting disease-free survival (*n* = 38)

	Univariate			Multivariate		
	RR	95 % CI	*P*-value	RR	95 % CI	*P*-value
Age	1.149	0.3154 – 4.190	0.8328	1.162	0.1601 – 4.622	0.8607
FIGO stage	1.877	0.4963 – 7.102	0.3535	2.524	0.2284 – 27.89	0.4501
Lymph node metastasis	1.937	0.5174 – 7.248	0.3263	1.197	0.4152 – 8.85	0.4044
Parametrium invasion	3.155	0.9579 – 11.76	0.0681	1.716	0.0402 – 8.416	0.691
Margin positive	3.884	0.3645 – 685.0	0.1581	1.604	0.03867 – 10.06	0.7392
Primary tumor > 4 cm	0.3786	0.05758 – 1.266	0.1609	0.3811	0.2314 – 29.76	0.4362
Serum SCC antigen > 5.0 ng/mL	0.6878	0.1982 – 2.365	0.5545	0.2663	0.04308 – 1.646	0.1545
LVSI	8.698	1.508 – 18.00	0.011*	9.764	0.7944 – 120.0	0.07506
Shrinkage rate ≦ 50 %	6.098	2.328 – 37.18	0.0021*	12.14	1.023 – 144.1	0.04794*

*RR* Relative risk, *CI* Confidence interval, Univariate analysis, Log rank test; Multivariate analysis, Cox proportional hazard model; *significant *p*-value

### Analysis of expression profiles of clinical samples

**Fig. 1:**
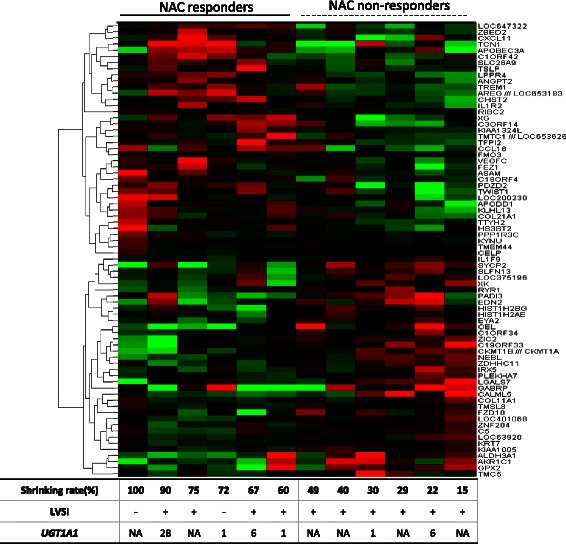
Expression pattern of discriminating genes of post-NAC tumors between responders (shrinkage rate ≧50 %) and non-responders (shrinkage rate < 50 %) among 12 LACC patients. The listed genes were extracted by comparative analysis using the SAMROC method with a *p*-value <0.001. Vertical columns represent individual samples, and the table at the bottom provides data on the shrinkage rate, LVSI, and *UGT1A1* polymorphism. Concerning *UGT1A1* polymorphism, 1, 6, and NA indicate “*UGT1A1* *1/*1 (UGT1A1-wild),” “heterozygotic polymorphism of UGT1A1 *1/*6,” and “not available” respectively

**Fig. 2:**
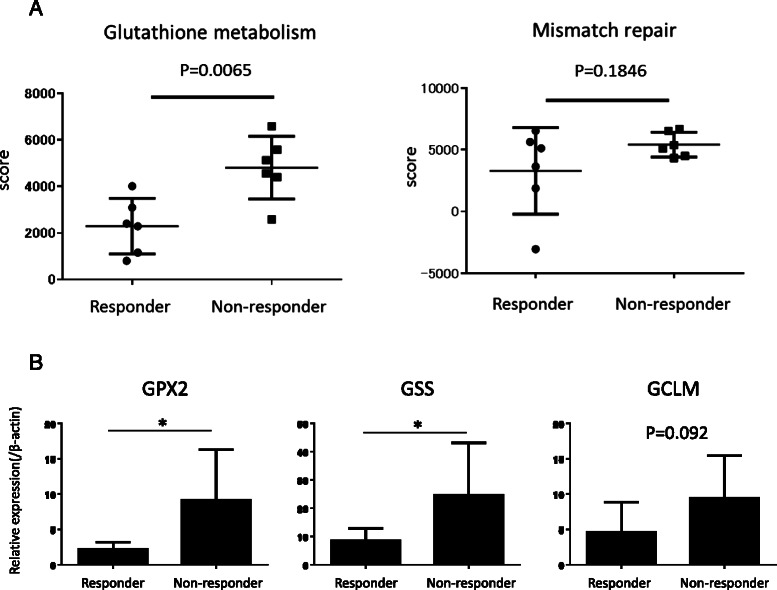
**a** Comparison of ssGSEA scores of glutathione metabolism and mismatch repair pathways between NAC-responders (*n* = 6) and non-responders (*n* = 6). The Mann–Whitney U test was used for statistical analysis of the two groups. **b** Relative expressions of GMP genes, *GPX2*, *GSS*, and *GCLM*, were compared between NAC-responders and non-responders based on quantitative PCR

Gene expression microarrays of 12 post-NAC tumors were analyzed to determine the representative signature of chemo-susceptibility in LACC, in order to compare NAC responders (shrinkage rate≧50 %, *n* = 6) with non-responders (rate <50 %, *n* = 6). SAMROC analysis detected 35 genes that were significantly up-regulated in non-responders (*p* < 0.001, Fig. 1), including drug-metabolism-related molecules such as ALDH3A1 (aldehyde dehydrogenase 3 family, member A1) and GPX2 (glutathione peroxidase 2). GSEA analysis revealed that metabolism-related and DNA repair system-related pathways were significantly up-regulated in non-responders (Table 3). The constitutive genes of the glutathione metabolic pathway (GMP) and mismatch repair pathway are shown in Additional file 4: Table S2. In ssGSEA analysis, the GMP score was significantly higher in non-responders (*p* < 0.01), while there was no significant difference in the mismatch repair pathway score (*p* = 0.1846, Fig. 2a). The prominent expression of GMP genes, *GPX2*, *GSS*, and *GCLM*, was confirmed in NAC-responders by qPCR (Fig. 2b).

**Table 3:** Genomic comparison between NAC-responders and non-responders. Enriched KEGG pathways in non-responders with shrinkage rates ≦ 50 %

KEGG pathway	Number of genes	NES	*p*-value	*q*-value
Metabolism-related				
METABOLISM_OF_XENOBIOTICS_BY_CYTOCHROME_P450	34	2.021684	<0.01	<0.001
DRUG_METABOLISM_CYTOCHROME_P450	36	1.987058	<0.01	0.002063
PROPANOATE_METABOLISM	29	1.925244	<0.01	0.001375
VALINE_LEUCINE_AND_ISOLEUCINE_DEGRADATION	41	1.920898	<0.01	0.001179
BUTANOATE_METABOLISM	26	1.873636	<0.01	0.003011
GLUTATHIONE_METABOLISM	42	1.652794	<0.01	0.031592
DNA repair system-related				
MISMATCH_REPAIR	21	1.935372	<0.01	0.00165
HOMOLOGOUS_RECOMBINATION	23	1.754282	<0.01	0.009237
BASE_EXCISION_REPAIR	33	1.62332	<0.01	0.041855
NUCLEOTIDE_EXCISION_REPAIR	42	1.588174	<0.01	0.042931
Others				
DNA_REPLICATION	33	2.245259	<0.01	<0.001
CELL_CYCLE	107	2.148499	<0.01	<0.001
ALDOSTERONE_REGULATED_SODIUM_REABSORPTION	25	1.668492	0.019608	0.027948
GLYCOSYLPHOSPHATIDYLINOSITOL_GPI_ANCHOR	23	1.597785	0.017544	0.042713

*KEGG* Kyoto Encyclopedia of Genes and Genomes Database, *NES* Normalized Enrichment Score

### GMP score shows a positive correlation with resistance to CPT-11

Using published COSMIC gene expression profiles of 57 SCC cell lines with half maximal inhibitory concentrations (IC50 values) for various anticancer drugs, ssGSEA analysis was performed. GMP scores exhibited a positive correlation with IC50 values for CPT-11 (r = 0.32, *p* < 0.05, Additional file 2: Figure S2A), but not with cisplatin. ssGSEA also showed that GMP scores of HCT-116 cells were significantly higher in CPT-11-resistant derivatives (*p* < 0.05, Additional file 2: Figure S2B). These results indicated that the GMP score could be a potent marker of resistance to CPT-11.

**Fig. 3:**
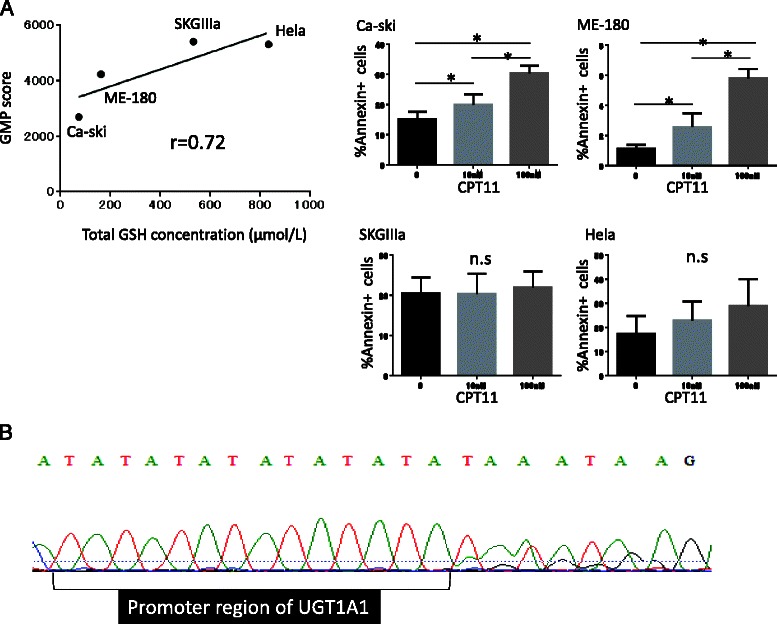
Genomic analysis of cervical cancer cell lines. **a** *Left:* Correlation analysis of GMP scores with intracellular GSH concentrations within 4 cervical cancer cell lines (*n* = 5). GMP scores were correlated with the total GSH concentration (r = 0.72). *Right:* Apoptosis was induced in each cell line in the presence of several concentrations of CPT-11 (0, 10, or 100 μM). *: *p* < 0.05. n.s.: not significant. **b** Targeted sequencing of the UGT1A1 gene in SKGIIIa. UGT1A1*28 polymorphism is designated as two base pair insertions of TA in the TATA box within the promoter region of the UGT1A1 gene. The A(TA)6TAA sequence in the wild-type allele become A(TA)7TAA in UGT1A1*28 polymorphism. Heterozygous UGT1A1*28 polymorphism exhibited waveform distortion downstream of the promoter region of the gene

To confirm the validity of the GMP score, the total intracellular glutathione concentration (GSH) resulting from glutathione metabolism was assessed in the 4 cultured cervical cancer cell lines. There was a positive correlation between GSH and GMP (r = 0.72), and, under treatment with CPT-11, apoptosis was induced in Ca-ski and ME-180 cells with low GMP and/or GSH scores in a dose-dependent manner (Fig. 3a). Furthermore, GPX2 and GCLM were more highly expressed in Hela and SKGIIIa cells, along with a high GSH concentration (Additional file 2: Figure S2C).

SKGIIIa exhibited a high GMP score, although it was designated as CPT-11-sensitive (Additional file 2: Figure S2A). Interestingly, *UGT1A1* polymorphism analysis revealed the presence of UGT1A1*28 heterozygotic polymorphism in SKGIIIa cells (Fig. 3b), indicating the contribution of host *UGT1A1* polymorphisms to the susceptibility of tumors to CPT-11.

### Clinical impact of UGT1A1 polymorphism

*UGT1A1* genotyping of patients’ blood samples led to the detection of heterozygotic polymorphisms (*1/*6, *1/*28) in 11 patients (48 %, UGT1A1-poly), while almost half of the patients were *UGT1A1* *1/*1 (UGT1A1-wild), and there were no patients with homozygotic (*6/*6, *28/*28) or compound heterozygotic (*6/*28) polymorphisms in this study. The tumor size was significantly reduced by NAC in UGT1A1-poly patients (77.5 %) compared with UGT1A1-wild patients (49.5 %, *p* < 0.05), although there were no differences in severe adverse events between them (Table 4).

**Table 4:** Association between UGT1A1 genotypes and either prognostic factors or Grade 3 to 4 toxicities (*n* = 23)

UGT1A1 genotypes	*1/*6 or*1/*28	*1/*1	*p*
	*n* = 11	*n* = 12	
Prognostic factors			
LVSI	5 (45 %)	6 (50 %)	0.827
Shrinkage rate (median +/− SD %)	77.5 +/− 26.8	49.5 +/− 24.1	0.028*
G3/4 Toxicities			
Neutropenia	6 (54.5 %)	8 (66.7 %)	0.552
Leukopenia	4 (36.4 %)	3 (25.0 %)	0.554
Anemia	2 (18.2 %)	1 (8.3 %)	0.484
Thrombocytopenia	1 (9.1 %)	0	0.286
Diarrhea	2 (18.2 %)	1 (8.3 %)	0.484
Vomiting	1 (9.1 %)	0	0.286

*significant *p*-value

## Discussion

The superiority of concurrent chemoradiation therapy (CCRT) over surgical excision for LACC due to its more favorable local control and reduced adverse event rates was described in a recent report [17], and CCRT is now designated as a standard therapy for LACC. However, CCRT has several difficulties in terms of preserving the ovarian function, reducing late radiation-based adverse effects, and managing pelvic lesions which remain or recur after CCRT [18]. Thus, a therapeutically intensive alternative has been investigated, and NAC-RH has been designated as a reasonable alternative for LACC in terms of not only avoiding primary radiation therapy, but also the therapeutic intensity [19]. In this study, the response rate to NAC using CPT-11 and NDP was 76.3 %, consistent with a previous report [10] and not inferior to the combination of cisplatin and CPT-11 [20] or paclitaxel [21], but nearly one quarter of the cases were non-responders, and those with tumor shrinkage rates following NAC below 50 % showed shorter PFS. This was in agreement with a previous report that non-responders had poor prognostic outcomes [22]. Thus, the shrinkage rate was an independent prognostic factor, and it should be predicted prior to NAC to reduce the risk of the main treatment being delayed without NAC benefits.

Pharmacogenomic analysis using gene expression microarrays with chemo-sensitivity profiles has successfully identified several “signatures” which are characteristic gene profiles in cancers resistant to specific anti-cancer drugs [5], and been employed to discover novel drugs effective against chemo-resistant cancers [23]. In this study, GSEA showed that a mismatch repair pathway, a well-known signal of platinum-resistance, was enriched in non-responders. ssGSEA, which quantitatively scores the activity of each “signature” pathway in every sample based on gene expression microarray [7], revealed that the GMP score significantly increased in non-responders, and that there was a positive correlation between GMP scores and IC50 values of CPT-11 among 57 SCC cell lines. These results suggest that GMP scores may represent the susceptibility of LACC to CPT-11.

CPT-11 shows a strong anti-tumor activity not only via the inhibition of DNA synthesis but also the inactivation of cystine transporters, leading to the accumulation of reactive oxygen species (ROS) in cancer cells [24]. As the presence of ROS is an apoptosis-inducing stress, this mechanism associating with glutathione synthesis may support the idea that GMP activity represents CPT-11 susceptibility. There is, however, a limitation of this study in that GMP was identified through the analysis of post-NAC tumors, and to apply this pathway as a predictive biomarker of NAC susceptibility, further validation based on pre-NAC tumors is necessary. Nonetheless, unsupervised hierarchical cluster analysis of a web-published SCC gene expression microarray, GSE 6213, which contains paired samples of identical patients before/after chemotherapy, revealed the following: The gene expression pattern was not markedly affected by chemotherapy, since cluster dendrograms did not divide samples of each individual patient before/after chemotherapy (Additional file 3: Figure S3). As the GMP score showed a strong positive correlation with the total glutathione concentration in SCC cells and SCC cells with low GMP scores showed high sensitivity to CPT-11, the GMP score might be expected to act as an NAC-susceptibility biomarker representing intracellular glutathione synthesis.

In contrast, patients with heterozygotic polymorphism of UGT1A1 *1/*6 or *1/*28 (UGT1A1-poly patients) showed a shrinkage rate > 50 % without a high incidence of G3/4 toxicities. The finding that SKGIIIa cells with a high GMP score showed *UGT1A1* polymorphisms and a high susceptibility to CPT-11 suggests that host *UGT1A1* polymorphism may constitute a complementary marker of CPT-11 susceptibility in an organ-specific manner. In colorectal cancers, FOLFIRI containing CPT-11 was more effective in patients with UGT1A1*28/*28 polymorphism than *UGT1A1*-wild patients accompanied with a decrease of SN38 glucuronidation in the liver [25]. Patients with homozygous polymorphism had a much higher AUC ratio of SN-38 than wild-type patients, whereas patients with heterozygous polymorphism had a slightly higher AUC ratio than wild-type patients [25]. This may be the reason why NAC containing CPT-11 showed a higher efficacy without marked toxicity in patients with heterozygous polymorphisms. The *UGT1A1* gene was picked up as one of the chemo-refractory signature genes in a genome-wide analysis [26], and UGT activation was observed in a colon cancer cell line which acquired resistance to CPT-11 [27]. We considered that the *UGT1A1* genotypes of cancerous tissues matched those of the host, although there has been no report to date on the correlation of *UGT1A1* polymorphisms between the tumor and host. Although further prospective studies are needed, NAC using CPT-11 in combination with *UGT1A1* genotyping might be performed more effectively and safely than other regimens without such markers.

## Conclusion

Based on comprehensive analysis, the present study suggests that the GMP score and heterozygotic *UGT1A1* polymorphisms may be complementary predictive markers of CPT-11 efficacy. Other than CPT-11, a comprehensive genomic analysis of both host and cancerous tissue might facilitate the establishment of a novel tailored therapy for LACC patients that exhibits high-level efficacy and feasibility.

## Additional files

Additional file 1: Figure S1. Kaplan-Meier curve for comparison of disease free survival according to prognostic parameters, stage, lymph node metastasis, LVSI, or shrinking rate. (PPT 124 kb)
Additional file 2: Figure S2. GMP scores in cancer cell lines. (A) Correlation of IC50 values of CPT-11 between 57 SCC cell lines with GMP scores. GMP scores were calculated by ssGSEA method. GMP scores significantly correlated with IC50 values of CPT-11 (r = 0.32, *p* = 0.016). Dotted black line exhibits average value of IC50. High IC50 value means to be resistant with drug. GMP, Glutathione Metabolism Pathway; IC50, half maximal Inhibitory concentration. (B) Comparison of GMP score between HCT-116 cell and CPT-11 resistant derivative. CPT-11 resistant cell exhibited significantly higher GMP score (*p* = 0.016). These microarray data were deposited to Array Express as E-MEXP-1171(http://www.ebi.ac.uk/arrayexpress/). (C) Relative expression of GMP genes, *GPX2*, *GSS*, and *GCLM*, in cervical cancer cell lines. (PPT 149 kb)
Additional file 3: Figure S3. Unsupervised hierarchical cluster analysis of a web-published SCC gene expression microarray, GSE 6213 which contains paired samples of identical patient before/after chemotherapy. Cluster dendrogram drawn by pvclust methods using R software exhibited gene expression pattern was not remarkably affected during chemotherapy. Four digits followed by X are case specific ID. Pre means samples harvested before treatment and post means samples harvested after treatment. Values on the edges of the clustering are p-values (%). Red values are AU (Approximate Unbiased) p-values, and green values are BP (Bootstrap Probability) values. Higher AU values exhibits stronger connection of clusters. (PPT 88 kb)
Additional file 4: Table S1. Clinical information of microarray samples of cancerous tissue. SCC: squamous cell carcinoma. NA: not available. **Table S2**. Gene lists of featured pathways drawn from MSigDB (The Molecular Signatures Database). (A) Glutathione Metabolism Pathway; (B) Mismatch Repair Pathway. **Table S3**. GMP scores of 57 SCC cell lines calculated by ssGSEA. IC 50 means 50 % inhibitory concentration of camptothecin, downloaded from COSMIC. (DOCX 33 kb)

## Abbreviations

NAC: Neoadjuvant chemotherapy
SCC: Squamous cell carcinoma
UGT1A1: UDP glucuronosyltransferase 1 family, polypeptide A1
LACC: Locally advanced cervical cancer
RH: Radical hysterectomy
GMP: Glutathione metabolic pathway
GSEA: Gene set enrichment analysis
PFS: Progression-free survival
LVSI: Lymphovascular invasion
IC50: Half maximal inhibitory concentration
