# Supplementary figures and images for: Genomic profile predicts the efficacy of neoadjuvant chemotherapy for cervical cancer patients
Source: BMC Cancer. 2015 Oct 19;15:739. doi: 10.1186/s12885-015-1703-1 (PMC4612400; doi:10.1186/s12885-015-1703-1)

## Slide 1
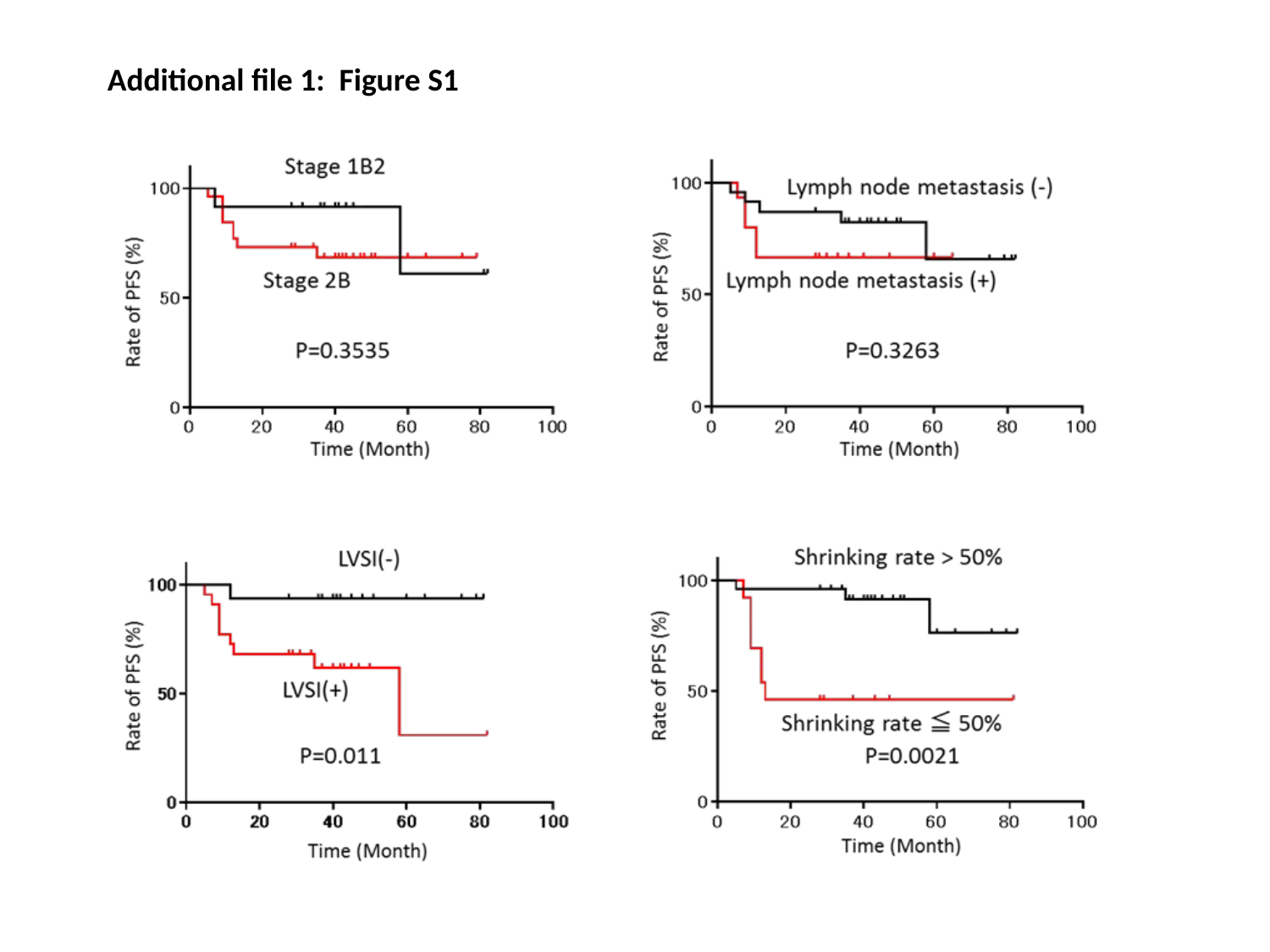

Additional file 1: Figure S1

Supplement: Additional file 1: Figure S1. — Kaplan-Meier curve for comparison of disease free survival according to prognostic parameters, stage, lymph node metastasis, LVSI, or shrinking rate. (PPT 124 kb) [file 12885_2015_1703_MOESM1_ESM.ppt]

## Slide 1
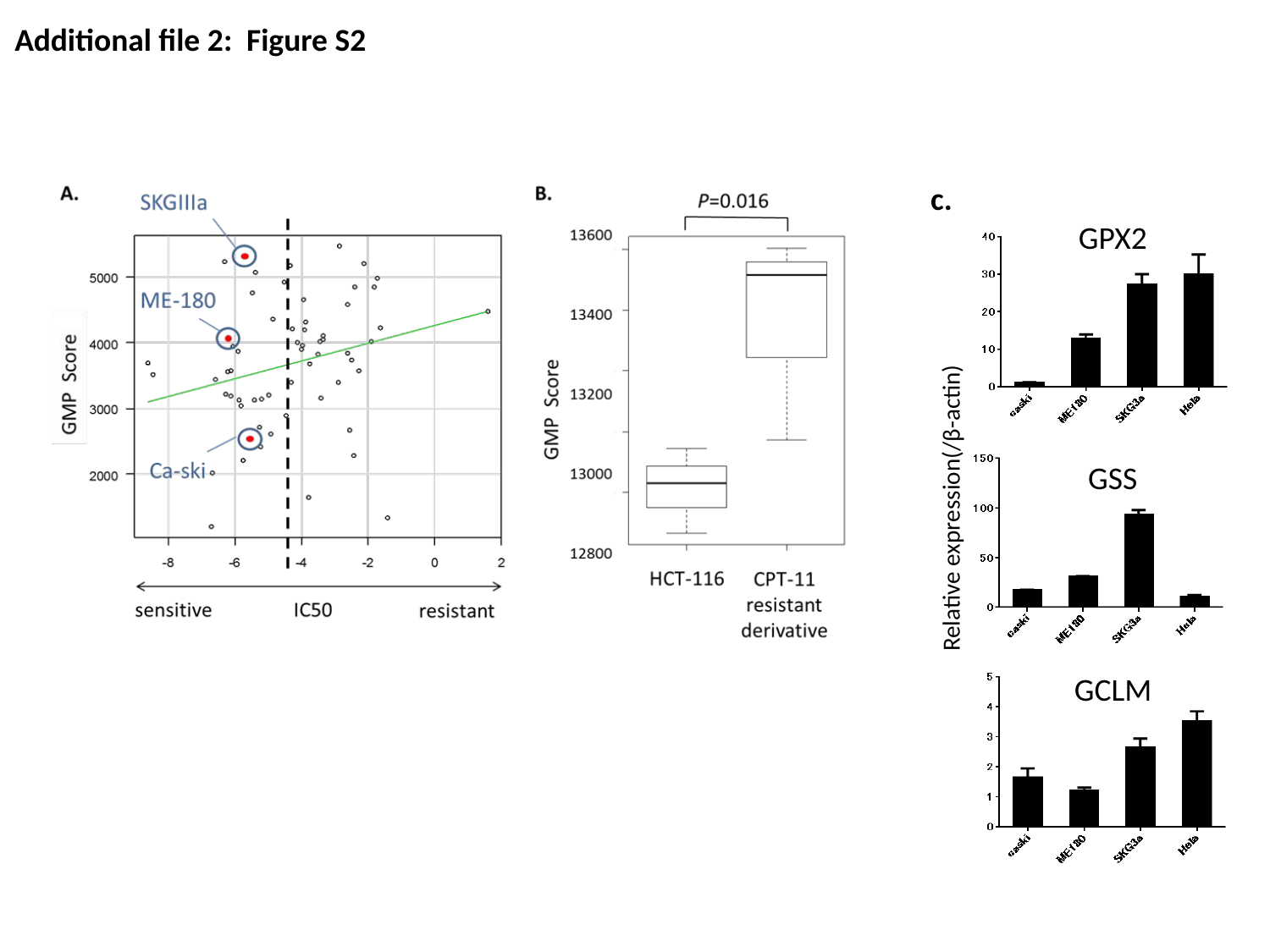

Additional file 2: Figure S2
c.
GPX2
GSS
Relative expression(/β-actin)
GCLM

Supplement: Additional file 2: Figure S2. — GMP scores in cancer cell lines. (A) Correlation of IC50 values of CPT-11 between 57 SCC cell lines with GMP scores. GMP scores were calculated by ssGSEA method. GMP scores significantly correlated with IC50 values of CPT-11 (r = 0.32, p = 0.016). Dotted black line exhibits average value of IC50. High IC50 value means to be resistant with drug. GMP, Glutathione Metabolism Pathway; IC50, half maximal Inhibitory concentration. (B) Comparison of GMP score between HCT-116 cell and CPT-11 resistant derivative. CPT-11 resistant cell exhibited significantly higher GMP score (p = 0.016). These microarray data were deposited to Array Express as E-MEXP-1171(http://www.ebi.ac.uk/arrayexpress/). (C) Relative expression of GMP genes, GPX2, GSS, and GCLM, in cervical cancer cell lines. (PPT 149 kb) [file 12885_2015_1703_MOESM2_ESM.ppt]
