# Supplementary material for: Genomic profile predicts the efficacy of neoadjuvant chemotherapy for cervical cancer patients
Source: BMC Cancer. 2015 Oct 19;15:739. doi: 10.1186/s12885-015-1703-1 (PMC4612400; doi:10.1186/s12885-015-1703-1)
Supplement: Additional file 3: Figure S3. — Unsupervised hierarchical cluster analysis of a web-published SCC gene expression microarray, GSE 6213 which contains paired samples of identical patient before/after chemotherapy. Cluster dendrogram drawn by pvclust methods using R software exhibited gene expression pattern was not remarkably affected during chemotherapy. Four digits followed by X are case specific ID. Pre means samples harvested before treatment and post means samples harvested after treatment. Values on the edges of the clustering are p-values (%). Red values are AU (Approximate Unbiased) p-values, and green values are BP (Bootstrap Probability) values. Higher AU values exhibits stronger connection of clusters. (PPT 88 kb) [file 12885_2015_1703_MOESM3_ESM.ppt]

## Slide 1
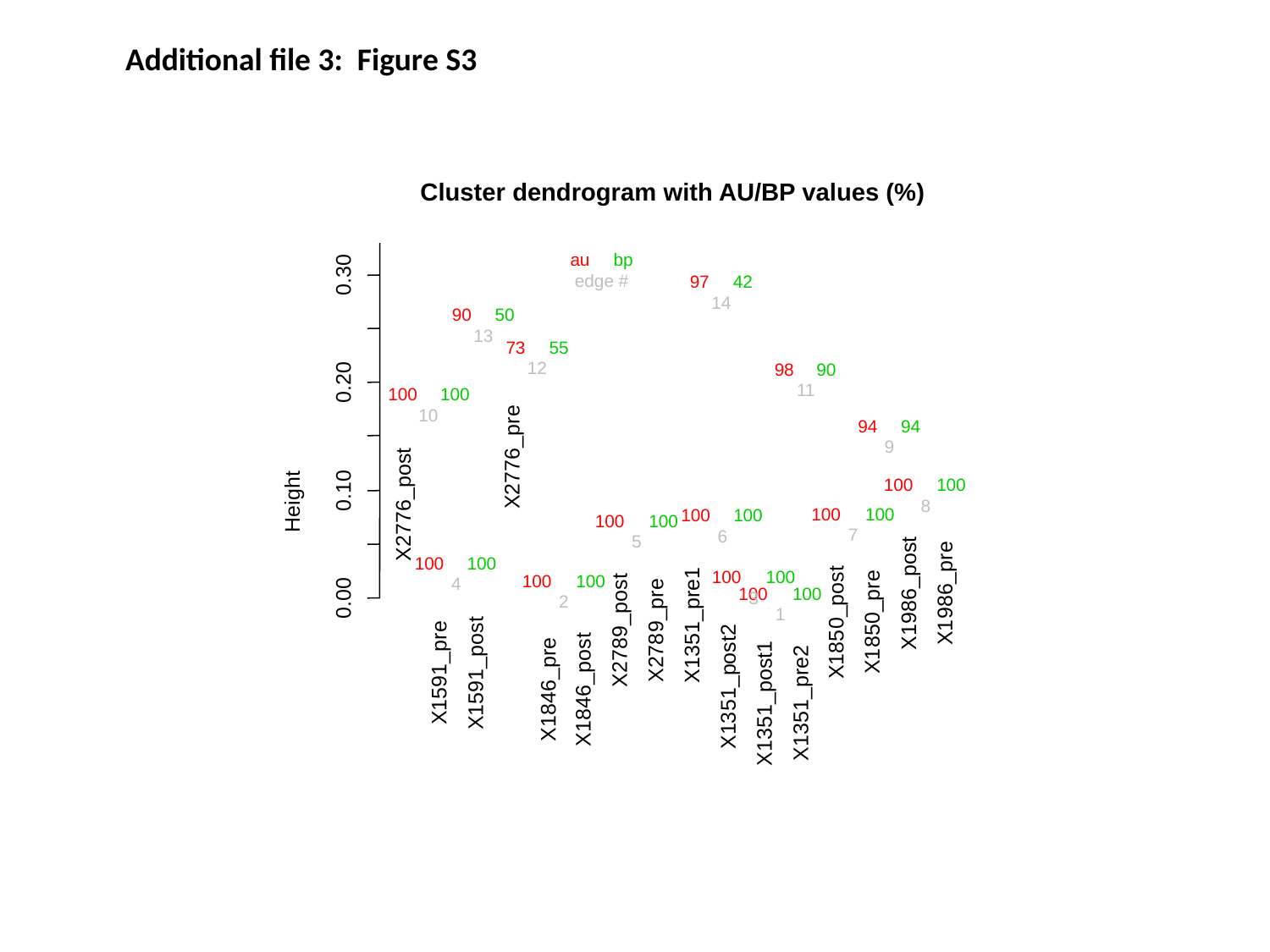

Additional file 3: Figure S3
Cluster dendrogram with AU/BP values (%)
au
bp
0.30
edge #
97
42
14
90
50
13
73
55
12
98
90
0.20
11
100
100
10
94
94
9
X2776_pre
100
100
0.10
Height
X2776_post
8
100
100
100
100
100
100
7
6
5
100
100
100
100
100
100
4
X1986_pre
X1986_post
100
100
0.00
3
2
1
X1850_pre
X1850_post
X1351_pre1
X2789_pre
X2789_post
X1591_pre
X1591_post
X1351_post2
X1846_pre
X1846_post
X1351_pre2
X1351_post1
