# Supplementary material for: Genomic profile predicts the efficacy of neoadjuvant chemotherapy for cervical cancer patients
Source: BMC Cancer. 2015 Oct 19;15:739. doi: 10.1186/s12885-015-1703-1 (PMC4612400; doi:10.1186/s12885-015-1703-1)
Supplement: Additional file 4: Table S1. — Clinical information of microarray samples of cancerous tissue. SCC: squamous cell carcinoma. NA: not available. Table S2. Gene lists of featured pathways drawn from MSigDB (The Molecular Signatures Database). (A) Glutathione Metabolism Pathway; (B) Mismatch Repair Pathway. Table S3. GMP scores of 57 SCC cell lines calculated by ssGSEA. IC 50 means 50 % inhibitory concentration of camptothecin, downloaded from COSMIC. (DOCX 33 kb) [file 12885_2015_1703_MOESM4_ESM.docx]

**Additional file 4**

***Genomic profile predicts the efficacy of neoadjuvant chemotherapy for cervical cancer patients***

Horikawa Naoki, Tsukasa Baba, Noriomi Matsumura, Ryusuke Murakami, Kaoru Abiko, Junzo Hamanishi, Ken Yamaguchi, Msafumi Koshiyama, Yumiko Yoshioka, and Ikuo Konishi

Department of Gynecology and Obstetrics, Kyoto University Graduate School of Medicine, Kyoto, JAPAN

**Correspondence:**

54 Shogoin Kawahara-cho, Sakyo-ku, Kyoto, Kyoto 606-8507, JAPAN

Phone; 81-75-751-3269, Fax; 81-75-761-3967, email; [babatsu@kuhp.kyoto-u.ac.jp](mailto:babatsu@kuhp.kyoto-u.ac.jp)

**Supplementary Material and Methods**

*Real Time Quantitative PCR.*

Total RNA was extracted from clinical samples and cervical cancer cell lines using the RNeasy® Mini Kit (QIAGEN, Tokyo, Japan). To monitor gene expression, quantitative reverse transcriptase (RT)-PCR amplification of target genes and beta-catenin mRNAs was done using the Light Cycler 480-II (Roche, Basel, Switzerland) and a Dual Color Hydrolysis Universal Probe System (Roche). The primers are listed as below, which were obtained from the Universal Probe Library Assay Design Center (Roche). Cycling parameters were 95^o^C for 10 seconds followed by 40 cycles of 95^o^C for 5 seconds and 60^o^C for 30 seconds, followed by a dissociation cycle of 95^o^C for 15 seconds, 60^o^C for 20 seconds, and 95^o^C for 15 seconds. The relative expression of target genes was estimated by dividing the threshold cycle (CT) value of target genes by the *beta-actin* CT values.

Primer sequences

*Gene Primer sequence*

*beta-actin Forward Primer: 5’ – CCA ACC GCG AGA AGG TGA – 3’*

*Reverse Primer: 5’ – CCA GAG GCG TAC AGG GAT AG – 3’*

*GPX2 Forward Primer: 5’ – TCT ATG ACC TCA GTG CCA TCA －3’*

*Reverse Primer: 5’ – TGT TCA GGA TCT CCT CAT TCT G – 3’*

*GSS Forward Primer: 5’ – CCT GCT AGT GGA TGC TGT CA – 3’*

*Reverse Primer: 5’ – TCA TCC TGT TTG ATG GTG CT – 3’*

*GCLM Forward Primer: 5’ – GTT GGA ACA GCT GTA TCA GTG G – 3’*

*Reverse Primer: 5’ – CAG TCA AAT CTG GTG GCA TC – 3’*

*Apoptosis Detection Assay.*

Cervical cancer cells were placed into 6 cm dishes and cultured until they were 80% confluent. The cell culture medium was replaced with fresh medium containing 0, 10, or 100 μM CPT-11 (Sigma Aldrich) and incubated for 24hr. Cells were harvested by trypsinization for apoptosis detection by flow cytometry. Following washing with phosphate-buffered saline, cells were resuspended in 100μL of 1× annexin-V binding buffer (BD Pharmingen, Inc.). Next, 5μL of annexin-V were added to the tubes and incubated for 10 minutes at 4°C in the dark. Cells were washed with 250μL of 1× annexin-V binding buffer and then resuspended in 200μL of 1× annexin-V binding buffer followed by filtration through a 70-μm nylon filter (Becton Dickinson Labware, Franklin Lakes, NJ). The samples were placed on ice and analyzed by FACSCalibur (Becton Dickinson). The data were analyzed using FlowJo v.7.6.3 (Tree Star, Ashland, OR).

*Microarray Data.*

Microarray data from 12 cervical cancer can be obtained at the Gene Expression Omnibus website (GSE70035, http://www.ncbi.nlm.nih.gov/geo/).

**Table S1:** Clinical information of microarray samples of cancerous tissue. SCC: squamous cell carcinoma. NA: not available.

|  | responder | | | | | | non-responder | | | | | |
| --- | --- | --- | --- | --- | --- | --- | --- | --- | --- | --- | --- | --- |
| shrinking rate(%) | 100 | 90 | 75 | 72 | 67 | 60 | 49 | 40 | 30 | 29 | 22 | 15 |
| age | 57 | 61 | 25 | 65 | 31 | 36 | 57 | 65 | 58 | 43 | 61 | 67 |
| FIGO stage | 2b | 1b2 | 1b2 | 2b | 1b2 | 2b | 2b | 2b | 2b | 2b | 2b | 1b2 |
| histology | SCC | SCC | SCC | SCC | SCC | SCC | SCC | SCC | SCC | SCC | SCC | SCC |
| LVSI | - | + | + | - | + | + | + | + | + | + | + | + |
| regime | CPT11/NDP | CPT11/NDP | CPT11/NDP | CPT11/NDP | CPT11/NDP | CPT11/NDP | CPT11/NDP | CPT11/NDP | CPT11/NDP | CPT11/NDP | CPT11/NDP | CPT11/NDP |
| course of NAC | 2 | 2 | 3 | 2 | 2 | 2 | 2 | 2 | 2 | 2 | 2 | 3 |
| Recurrence | - | - | - | - | - | - | vaginal stump | Paraaortic LN | Supraclavicular LN | - | - | - |
| Disease free intervals (month) | 52 | 50 | 48 | 29 | 42 | 45 | 13 | 12 | 9 | 52 | 32 | 39 |
| GMP score | 1273.9 | 1857.5 | 3174.7 | 4115.2 | 2372.7 | 2484.7 | 4566 | 6772.179114 | 2687.662217 | 5675.052634 | 4553.753533 | 5328.2 |
| UGT1A1  genotype | NA | *1/*28 | NA | *1/*1 | *1/*6 | *1/*1 | NA | NA | *1/*1 | NA | *1/*6 | NA |

LN: lymph nodes. NA: Not available. SCC: squamous cell carcinoma

**Table S2** Gene lists of featured pathways drawn from MSigDB (The Molecular Signatures Database). (A) Glutathione Metabolism Pathway; (B) Mismatch Repair Pathway.

(A)

| SRM | GPX6 | IDH2 |
| --- | --- | --- |
| GGT1 | GSTM4 | GPX4 |
| GSTP1 | GGCT | IDH1 |
| GSTT2 | GSTM3 | OPLAH |
| GSTT1 | GSTM2 | GCLM |
| GSTZ1 | GSTM5 | GGT6 |
| RRM2B | GSTA1 | ANPEP |
| SMS | GSTA2 | GGT7 |
| PGD | GSR | GSTO2 |
| GSTO1 | GSS | GGT5 |
| GSTA5 | RRM1 | G6PD |
| MGST2 | RRM2 | ODC1 |
| LAP3 | GCLC | GPX1 |
| MGST1 | GSTK1 | GPX2 |
| MGST3 | GPX5 | GPX3 |
| GSTA3 | TXNDC12 | GPX7 |
| GSTM1 | GSTA4 |  |

(B)

| MLH3 | LIG1 | MSH3 |
| --- | --- | --- |
| POLD1 | RFC5 | POLD4 |
| MLH1 | RPA1 | PMS2 |
| POLD2 | MSH6 | RFC4 |
| RFC1 | RPA3 | SSBP1 |
| MSH2 | POLD3 | RPA4 |
| RFC3 | RPA2 | EXO1 |
| RFC2 | PCNA |  |

**Table S3:** GMP scores of 57 SCC cell lines calculated by ssGSEA. IC 50 means 50% inhibitory concentration of camptothecin, downloaded from COSMIC.

| Cell line | IC50 | GMP score | Cell line | IC50 | GMP score |
| --- | --- | --- | --- | --- | --- |
| HT-3 | -8.4596 | 3516.864355 | KYSE-270 | -5.2547 | 2713.121617 |
| FADU | -6.2643 | 3223.235551 | KYSE-510 | -4.9977 | 3200.453279 |
| SAS | -6.6922 | 2011.825496 | TE-5 | -4.909 | 2604.43141 |
| HN | -6.5719 | 3445.615284 | KYSE-180 | -4.3291 | 5178.694182 |
| CAL-27 | -6.2182 | 3561.649969 | TE-11 | -4.1146 | 3997.538917 |
| Ca9-22 | -6.1145 | 3185.720972 | TE-9 | -3.7442 | 3680.551578 |
| RPMI-2650 | -6.1117 | 3572.085292 | KYSE-450 | -2.896 | 3391.424395 |
| HSC-3 | -5.8041 | 3044.708953 | TE-1 | -3.3493 | 4045.55649 |
| KOSC-2 | -5.4277 | 3132.864047 | KYSE-140 | -2.2834 | 3568.857129 |
| SKG-IIIa | -5.3856 | 5074.036221 | COLO-680N | -2.6249 | 4573.618027 |
| Ca-Ski | -5.7495 | 2200.77111 | KYSE-410 | -3.5086 | 3818.148065 |
| BHY | -5.2381 | 2413.136929 | KYSE-70 | -2.3845 | 4842.502245 |
| HCE-T | -5.2086 | 3147.02726 | KYSE-520 | -2.4942 | 3739.875981 |
| CAL-33 | -6.0638 | 3940.954216 | KYSE-150 | -1.4026 | 1337.957224 |
| C-33-A | -6.7127 | 1204.912823 | TE-12 | 1.5988 | 4480.639932 |
| KNS-62 | -4.8664 | 4364.027524 | TE-6 | -1.7232 | 4987.108161 |
| SK-MES-1 | -4.5296 | 4915.572318 | TE-10 | -1.9132 | 4022.754432 |
| HSC-2 | -4.4485 | 2895.038302 | SW900 | -3.447 | 4011.470195 |
| Detroit562 | -4.3172 | 3395.964015 | SCC-9 | -3.4055 | 3159.216694 |
| NCI-H2170 | -4.2897 | 4205.904871 | LK-2 | -3.3372 | 4100.183023 |
| SCC-15 | -3.9863 | 3899.553729 | SCC-25 | -2.8695 | 5464.31896 |
| CAL-39 | -3.9551 | 3955.880183 | EPLC-272H | -2.6201 | 3838.841075 |
| SCC-4 | -3.9497 | 4656.492917 | OMC-1 | -2.5484 | 2663.001399 |
| SW954 | -3.8948 | 4195.289412 | SiHa | -2.4432 | 2277.102605 |
| SW962 | -3.8867 | 4314.940609 | BB49-HNC | -2.118 | 5203.001374 |
| BB30-HNC | -5.8641 | 3132.461488 | NCI-H520 | -1.8201 | 4846.93271 |
| NCI-H226 | -3.7948 | 1650.807495 | LB771-HNC | -1.6254 | 4226.802648 |
| HCE-4 | -6.3122 | 5237.341969 | ME_180 | -5.9175 | 3866.036542 |
| TE-8 | -5.4798 | 4753.66966 |  |  |  |
